# Effect of simethicone on visualization quality and capsule transit time during video capsule endoscopy in healthy dogs

**DOI:** 10.1093/jvimsj/aalag183

**Published:** 2026-08-26

**Authors:** Wooram Jeong, Gyu-Hyun Kim, Se-Hoon Kim, Min-Ok Ryu, Kyoung-Won Seo

**Affiliations:** Laboratory of Veterinary Internal Medicine, Department of Veterinary Clinical Science, College of Veterinary Medicine, Seoul National University, Seoul 08826, Republic of Korea; Laboratory of Veterinary Internal Medicine, Department of Veterinary Clinical Science, College of Veterinary Medicine, Seoul National University, Seoul 08826, Republic of Korea; Laboratory of Veterinary Internal Medicine, Department of Veterinary Clinical Science, College of Veterinary Medicine, Seoul National University, Seoul 08826, Republic of Korea; Laboratory of Veterinary Internal Medicine, Department of Veterinary Clinical Science, College of Veterinary Medicine, Seoul National University, Seoul 08826, Republic of Korea; Laboratory of Veterinary Internal Medicine, Department of Veterinary Clinical Science, College of Veterinary Medicine, Seoul National University, Seoul 08826, Republic of Korea

**Keywords:** capsule endoscopy, image, mucosa, simethicone, small bowel, small dog

## Abstract

**Background:**

Simethicone enhances visualization quality (VQ) during video capsule endoscopy (VCE) in humans, but its effects on VQ and capsule transit time (CTT) in dogs remain unclear.

**Hypothesis/Objectives:**

Evaluate the effects of simethicone on VQ and CTT in dogs undergoing VCE.

**Animals:**

Fifteen client-owned healthy dogs weighing 3-10 kg.

**Methods:**

Prospective, blinded, randomized crossover study. Each dog underwent VCE twice: once with 20 mg (1 mL) simethicone administered 30 minutes before capsule ingestion, and once with 1 mL normal saline as a control. Visualization quality was assessed based on the proportion of mucosa visualized (variable 1), the degree of obscuration by bubbles, debris, blood, or bile (variable 2), and their average (variable 3). Capsule transit time, including gastric transit time (GTT) and small bowel transit time (SBTT), and incomplete study rates also were evaluated.

**Results:**

Simethicone significantly improved VQ in the entire small bowel, especially in the proximal segment (*P* < .05 for all variables). However, no significant differences were observed between protocols in the distal small bowel and stomach. Gastric transit time and SBTT also were not significantly different between protocols. The risk of incomplete studies was not significantly affected by protocol, sex, body weight, or age.

**Conclusions and clinical importance:**

Simethicone had region-specific effects on VQ in dogs undergoing VCE, specifically enhancing VQ in the small intestine. These findings provide preliminary evidence of a potential role for simethicone in improving small bowel VQ.

## Introduction

Video capsule endoscopy (VCE) is a useful, non-invasive diagnostic modality that allows evaluating the gastrointestinal (GI) tract in dogs.^1–11^ It has been applied in various investigations of GI disorders, including GI bleeding of unknown origin, angiodysplasia, multiple gastric erosions, and chronic enteropathy.^1–10^ In human medicine, clinical guidelines for VCE provide useful information regarding its proper indications and optimize diagnostic outcomes by offering recommendations, including bowel preparation.^12^^,^^13^ However, such guidelines are absent in veterinary medicine, limiting the clinical application of VCE.

Visualization clarity of the mucosa is an important factor influencing diagnostic yield and may affect clinical decision-making.^14^^,^^15^ Bubbles are key obscuring materials that can significantly impair mucosal visualization.^16^ Suboptimal visualization quality (VQ) can compromise diagnostic accuracy by obscuring subtle mucosal lesions, potentially necessitating repeat examinations or additional invasive procedures. Among the factors affecting VQ, intraluminal bubbles and foam represent modifiable contributors that can be targeted by pharmacologic intervention. In human medicine, administering anti-foaming agents before VCE is recommended by current guidelines to address this specific component of visualization impairment and to enhance overall mucosal clarity.^13^^,^^14^^,^^16–20^ Simethicone, an effective antifoaming agent, significantly improves image quality by decreasing bubbles and foam within the GI tract in humans.^13^^,^^14^^,^^16–20^ Simethicone also has been used in veterinary practice in dogs undergoing VCE.^7^^,^^10^ However, its effect on VQ during VCE in dogs remains unclear, leaving bowel preparation protocols for veterinary use without a standardized, evidence-based foundation.

Although similarities between GI physiology in humans and dogs have supported the use of dogs in preclinical research, several clinically relevant species-specific differences (eg, length or diameter of intestine) persist,^21^^,^^22^ cautioning against the direct translation of GI data from humans to veterinary patients.^21^ These distinctions highlight a critical gap in the current literature and underscore the importance of generating foundational evidence for developing evidence-based VCE preparation guidelines specific for dogs.

Understanding simethicone effects on VQ during VCE in dogs is essential for establishing an evidence-based protocol that can improve clinical outcomes and enhance practitioners’ understanding of the characteristics and potential utility of this diagnostic tool. Accordingly, we aimed to evaluate simethicone administration effects on VQ and capsule transit time (CTT) during VCE in dogs. We explored whether simethicone administration was associated with incomplete studies and complications, and whether patient characteristics (eg, sex, body weight, and age) influence these outcomes.

## Materials and methods

### Dogs

Clinically healthy dogs weighing 3-10 kg were eligible for enrollment, because we aimed to evaluate the effect of simethicone specifically in small dogs to minimize interindividual differences in body weight that may influence CTT and risk of incomplete studies. Before enrollment, all dogs underwent a thorough clinical screening, including medical history, physical examination, blood tests (including complete blood count [CBC], biochemistry, and blood gas analysis), and abdominal radiography. Dogs with no evidence of concurrent disease based on these evaluations were defined as clinically healthy. Dogs were included only if they had no vomiting, diarrhea, or other GI signs for at least 1 month before enrollment. Dogs were excluded if they had a history of a chronic GI disorder, had undergone prior GI surgery, or were currently receiving medications known to affect GI motility.

### Video capsule endoscopy procedure

Video capsule endoscopy was performed using a commercially available MiroVET VC1000 capsule endoscope (Intromedic, Seoul, Republic of Korea; length: 23.0 mm, diameter: 9.5 mm, battery life: approximately 8 hours). The study comprised 2 protocols. In Protocol 1, 20 mg (1 mL) of simethicone (Gasocol suspension; TAEJOON PHARM, Seoul, Republic of Korea) was administered 30 minutes before capsule administration. In Protocol 2, an equivalent volume of normal saline (NS) was administered as a control. All dogs were fasted for >12 hours before the procedure. Additionally, ingestion of poorly digestible or hard foods, including bone-based foods, was restricted for 2-3 days before undergoing VCE. Each dog wore a vest containing an image receiver that was positioned over the dorsal area to collect data transmitted by the capsule. The vest was appropriately sized to fit each dog’s body conformation, ensuring comfort and avoiding excessive pressure. Before capsule administration, the recording system was tested to confirm proper functioning of the device. After capsule ingestion, each dog remained in a private space with its owner to minimize stress-related effects on GI motility. Dogs were allowed a gentle walk for ≤15 minutes per hour. Capsule progression through the GI tract was monitored at 1-h intervals using a receiver. The procedure was concluded either when the capsule reached the colon or when the device’s battery was depleted. Each dog underwent both protocols in randomized order, with a minimum washout period of 1 week between the procedures. The second examination only was performed if the dog remained clinically healthy and did not show GI signs during the washout period. The owners were instructed to discard the capsule upon its excretion in the feces and to record the date of excretion. All procedures were approved by the Institutional Animal Care and Use Committee of Seoul National University and conducted in accordance with relevant institutional and national guidelines for the care and use of laboratory animals. All owners signed a consent form before their dogs were enrolled.

### Outcome measures

Gastric transit time (GTT) was defined as the interval between the first captured images of the stomach and small intestine. In contrast, small bowel transit time (SBTT) was defined as the time elapsed between the first image of the small intestine and the point at which either the ileocolic valve was identified, or the colonic lumen became visible. For each dog, 20 and 40 images were extracted from the stomach and small intestine, respectively, at regular intervals determined by each dog’s transit time to ensure consistent sampling. Capture intervals were calculated by dividing the total GTT and SBTT by the number of images required for each region. Based on the calculated value, we selected the largest feasible time interval approximating the value from a set of predefined units. The available options for minute-based intervals included 1-10 minutes (1-min increments), 1.5 minutes, and 15 minutes. For second-based intervals, the options included 1-10 s (1-s increments), 15 s, and 30 s. For the stomach, 10 images were sequentially captured from the early portion of the gastric segment, whereas 10 other images were retrogradely captured from the transition point to the small intestine, which yielded a balanced representation of the entire gastric region. For the small intestine, 20 images were extracted from the proximal portion immediately after the gastric exit, whereas 20 other images were obtained from the distal portion just before the colon appeared, with all images sampled at the predefined intervals described above across the entire SBTT, ensuring even distribution throughout the small intestine. These 2 segments were designated as the proximal and distal small bowel.

**Figure 1:**
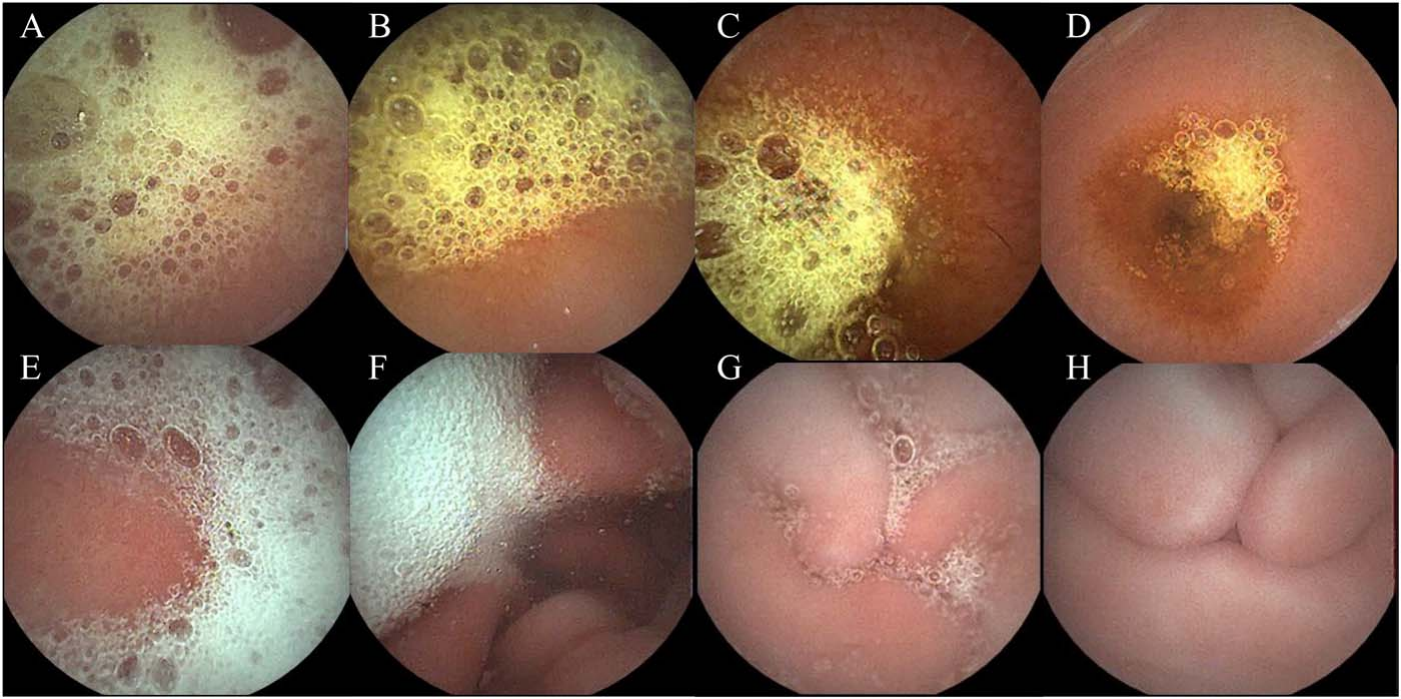
Video capsule endoscopic images with different scoring results. A-D, scored images based on the percentage of visualized mucosa (variable 1); A, score 0 (<25%); B, score 1 (≥25% and < 50%); C, score 2 (≥50% and < 75%); D, score 3 (≥75%). E-H, scored images based on the degree of obscuration by bubbles, debris, blood, and bile (variable 2); E, score 0 (≥50%); F, score 1 (≥25% and < 50%); G, score 2 (≥5% and < 25%); H, score 3 (<5%).

The VQ of images in the stomach and small intestine was assessed using a scoring system previously established in human medicine, with scores ranging from 0 to 3 based on 2 variables (Table 1, Figure 1).^15^ Variable 1 was defined as the proportion of mucosa visualized, whereas variable 2 was defined as the degree of obscuration by bubbles, debris, blood, or bile. These separate variables were applied to capture complementary aspects of VQ and to provide a more reliable evaluation. Furthermore, each image was scored again based on its average value (variable 3). The mean scores of the images were calculated for 4 anatomical regions: the stomach, as well as the entire small bowel, the proximal small bowel, and the distal small bowel. The score for the entire small bowel was obtained by combining data from the proximal and distal small bowel (40 images).

**Table 1:** Visualization scoring system of video capsule endoscopic images.

**Variable 1. The percentage of visualized mucosa**	
**Score 3**	≥75% (good)
**Score 2**	≥50% and <75% (adequate)
**Score 1**	≥25% and <50% (limited)
**Score 0**	<25% (poor)
**Variable 2. The degree of bubble, debris, blood, and bile**	
**Score 3**	<5%, no obscuration
**Score 2**	≥5% and <25%, mild obscuration
**Score 1**	≥25% and <50%, moderate obscuration
**Score 0**	≥50%, severe obscuration

Scores were determined by evaluating the percentage of visualized mucosa and the degree of obscuration by bubble, debris, and bile in each GI segment.

Two independent observers were trained to calibrate the scoring system and evaluate the extracted images. The most experienced observer, who had performed >30 capsule endoscopies, was designated as the chief observer, with the other observer assisting. For anonymization, each video was assigned a randomly generated identification number using Microsoft Excel (Microsoft, Redmond, WA, United States). Further, the files were encrypted by a staff member not involved in the study.

To evaluate the reliability of VQ assessment, different types of agreement analysis were performed. To assess inter-observer agreement, each observer independently evaluated the same image set extracted from each included dog. To assess intra-observer agreement, the chief observer re-scored the same images after a 1-month interval. To assess intra-patient agreement and validate the consistency of the image extraction method, the 2 observers independently extracted images from the same video recording. Subsequently, the extracted image sets were evaluated by a chief observer, with the VQ scores being compared to determine the reproducibility of the results.

For each procedure, the GTT and SBTT were derived from the CTT. We also performed between-protocol comparisons of the transit times to assess potential effects of simethicone administration on GI motility. If the battery was depleted within the stomach, the dog was excluded from the VQ and CTT analyses. However, in the case of an incomplete study of the small intestine, the VQ of the stomach and GTT were retained for between-protocol comparisons.

Complications related to VCE, including incomplete study, capsule retention, and post-procedure adverse effects, were recorded. An incomplete study was indicated by the capsule not reaching the colon before battery depletion. Capsule retention was defined as the capsule remaining in the GI tract for >2 weeks. Adverse effects, such as GI signs and anorexia, were monitored after each procedure.

### Statistical analysis

Between-protocol comparisons of VQ scores at different locations were performed using a paired *t-*test or a Wilcoxon signed-rank test, depending on the normality of data distribution as evaluated using the Shapiro–Wilk test. Further, between-protocol comparisons of GTT and SBTT were performed using the same statistical approach. Only paired data were included in the analysis of VQ and CTT. The intraclass correlation coefficient (ICC) was used to assess intra-observer, inter-observer, and intra-patient agreements. Values for ICC of 0.4, 0.4-0.75, and >0.75 reflected poor, fair-to-good, and excellent agreement, respectively.

Univariate logistic regression analyses were performed to identify risk factors for incomplete study. Each dog was classified as having experienced an incomplete study in at least one of the procedures or having completed both studies. Independent variables included sex as a binary variable, as well as body weight and age, which were dichotomized based on the mean or median value. Odds ratios (ORs) and the corresponding 95% CIs were calculated for all variables. Owing to the limited sample size, multivariable logistic regression was not performed. Statistical significance was set at *P* < .05. All statistical analyses were performed using SPSS software [version 29.0.2.0(20); IBM Corp., Armonk, NY].

## Results

### Dogs

Among the 19 eligible dogs, 2 dogs were withdrawn after the first protocol because of owner concerns about an incomplete study on the second protocol. Additionally, 2 dogs were excluded because of GI signs (1 with acute diarrhea and 1 with acute vomiting) observed before the first procedure. Accordingly, 15 dogs were included in the final analysis.

The median weight was 7 kg (range, 3.84-10 kg), and the mean age was 7.67 years (range, 2-11.9 years). Out of a total score of 9, the body condition scores were 3, 4, and 5 in 1, 9, and 5 dogs, respectively. There were 12 neutered males and 3 spayed females. None of the dogs had concurrent diseases related to GI motility, and no medication was administered before the tests. All dogs received 20 mg of simethicone using 3 mL syringe, corresponding to a dosage range of 2-5.21 mg/kg (median, 2.86 mg/kg).

### Visualization quality

Table 2 summarizes the mean visualization scores assessed by different variables and GI tract locations. In the stomach, no significant between-protocol differences were observed in any of these variables. In contrast, simethicone significantly improved the VQ scores in the small intestine. Specifically, simethicone significantly improved scores in the entire (*P* < .001 for all variables) and proximal small bowel (*P* = .02, .01, and .01 for variables 1, 2, and their average, respectively). No significant between-protocol differences were observed in any of the variables in the distal small bowel.

**Table 2:** Mean visualization scores assessed by different variables and locations of the gastrointestinal tract.

	Variable	Control (NS) (A)	Simethicone (B)	∆ Difference (A-B)	95% CI	*t*	*P*-value
**Stomach (*n* = 12)**	1	2.00 ± 0.75	1.59 ± 0.87	0.41 ± 0.64	0.00-0.82	2.195	.05
	2	1.34 ± 0.69	1.02 ± 0.64	0.32 ± 0.59	−0.06-0.69	1.867	.09
	3	1.67 ± 0.71	1.30 ± 0.75	0.36 ± 0.61	−0.02-0.75	2.08	.06
**Entire SB (*n* = 11)**	1	2.41 ± 0.31	2.74 ± 0.20	−0.33 ± 0.20	−0.46-0.19	−5.412	**<.001**
	2	1.63 ± 0.33	2.02 ± 0.31	−0.39 ± 0.22	−0.54-0.24	−5.87	**<.001**
	3	2.02 ± 0.31	2.38 ± 0.24	−0.36 ± 0.20	−0.49-0.22	−5.862	**<.001**
**Proximal SB**	1	2.25 ± 0.33	2.64 ± 0.37	−0.39 ± 0.45	−0.70--0.09	−2.892	**.02**
	2	1.47 ± 0.35	2.04 ± 0.55	−0.56 ± 0.56	−0.94-0.19	−3.326	**.01**
	3	1.86 ± 0.33	2.34 ± 0.45	−0.48 ± 0.50	−0.81-0.14	−3.193	**.01**
**Distal SB**	1	2.57 ± 0.42	2.83 ± 0.20	−0.26 ± 0.44	−0.55-0.03	−1.972	.08
	2	1.80 ± 0.43	2.01 ± 0.21	−0.21 ± 0.37	−0.46-0.03	−1.928	.08
	3	2.18 ± 0.41	2.42 ± 0.19	−0.24 ± 0.40	−0.50-0.03	−1.97	.08

The visualization scores are presented as mean ± SD. A paired *t*-test was used for between-protocol comparisons of all variables of the visualization score. Variable 1 indicates the proportion of mucosa visualized; variable 2, the degree of obscuration by bubbles, debris, blood, or bile; and variable 3, the mean of variables 1 and 2. *P*-values in bold are considered statistically significant.

Abbreviations: CI = confidence intervals; NS = normal saline; SB = small bowel; SD = standard deviation

**Figure 2:**
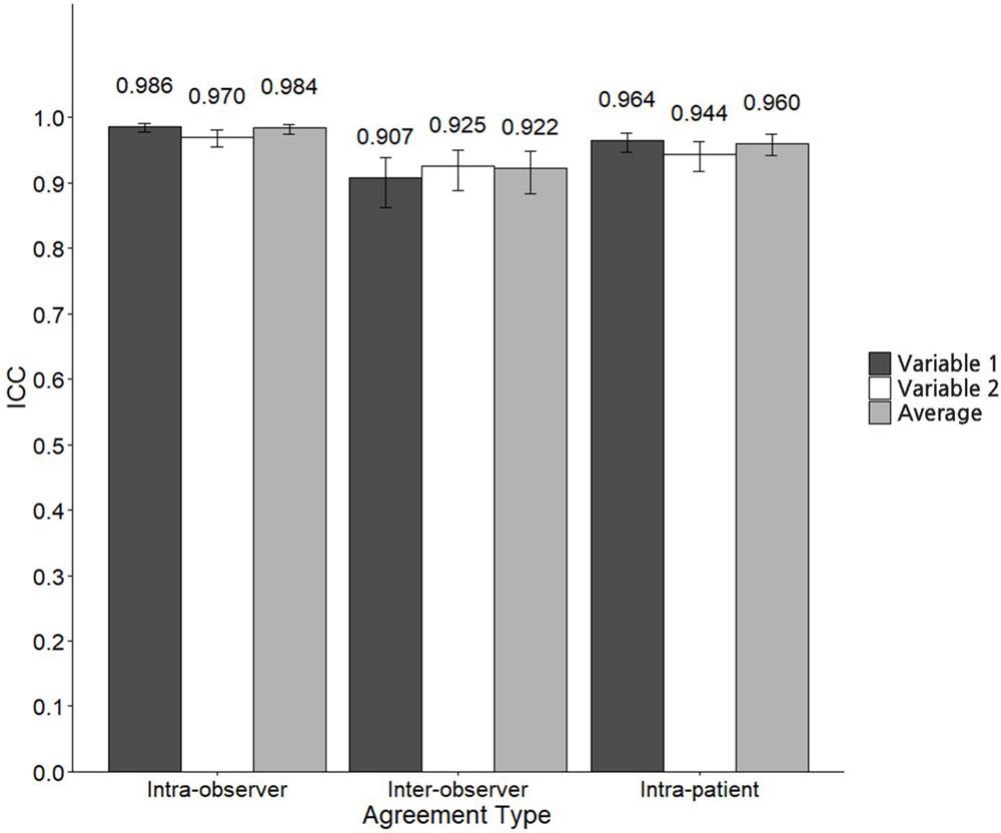
Intraclass correlation coefficients (ICC) for intra-observer, inter-observer, and intra-patient agreement across 3 visualization scoring metrics: Variable 1, variable 2, and their average.

The intra-observer, inter-observer, and intra-patient agreements were all excellent, as demonstrated by ICC values exceeding 0.90 across all comparisons (Figure 2). The ICC values for intra-observer agreement for variables 1, 2, and their average were 0.986 (95% CI, 0.978-0.991), 0.970 (95% CI, 0.954-0.980), and 0.984 (95% CI, 0.975-0.989), respectively. The ICC values for inter-observer agreement for variables 1, 2, and their average were 0.907 (95% CI, 0.862-0.938), 0.925 (95% CI, 0.888-0.950), and 0.922 (95% CI, 0.883-0.948), respectively. The ICC values for intra-patient agreement for variables 1, 2, and their average were 0.964 (95% CI, 0.947-0.976), 0.944 (95% CI, 0.917-0.963), and 0.960 (95% CI, 0.941-0.974), respectively.

### Capsule transit time

Table 3 presents the gastric and SBTTs for each protocol. The mean GTT for the control and simethicone protocols were 95.33 ± 103.67 (95% CI, 29.46-161.20) and 51.17 ± 34.61 (95% CI, 29.18-73.16), respectively. The mean SBTT for the control and simethicone protocols were 73.42 ± 44.20 (95% CI, 45.33-101.50) and 64.75 ± 31.72 (95% CI, 44.60-84.90), respectively. No significant between-group differences were observed in GTT (Z = –1.646, *P* = .1) or SBTT (t = 1.229, *P* = .25). Twelve dogs (paired data) were included in the GTT analysis and 11 dogs (paired data) in the SBTT analysis.

**Table 3:** Mean values of the gastric and small bowel transit times in each protocol.

	**Control (NS) (A)**	**Simethicone (B)**	**∆ Difference (A-B)**	**95% CI**	***t***	***P*-value**
**GTT (*n* = 12)**	95.33 ± 103.67	51.17 ± 34.61	44.17 ± 92.77	−14.77-103.11	−1.646^a^	.1^a^
**SBTT (*n* = 11)**	73.42 ± 44.20	64.75 ± 31.72	12.55 ± 33.86	−10.20-35.30	1.229	.25

The capsule transit time (minute) is presented as mean ± SD. A paired *t*-test or Wilcoxon signed rank test was used for between-protocol comparisons of the capsule transit time. *P*-values in bold are considered statistically significant.

Abbreviations: CI = confidence intervals; GTT = gastric transit time; NS = normal saline; SBTT = small bowel transit time.

^a^: Wilcoxon signed rank test used; Z-value is reported instead of *t*-value.

### Limitations and complications

Among 30 procedures, 5 (17%) were classified as incomplete owing to battery depletion before reaching the colon (Table 4). Battery depletion occurred in the stomach and small bowel in 3 and 2 procedures, respectively. Four dogs had at least 1 incomplete study. One dog weighing 10 kg experienced an incomplete study in both protocols, whereas the other 3 had incomplete studies in only 1 protocol (1 with simethicone and 2 with the control). The incomplete study rates were 20% and 13% for the control and simethicone protocol, respectively, with no significant between-protocol difference (OR, 0.62; 95% CI, 0.09-4.34; *P* = .63). Other analyses of risk factors, including sex (OR, 10.0; 95% CI, 0.58-171.20; *P* = .11), age (OR, 5.26; 95% CI, 0.40-68.95; *P* = .21), and body weight (OR, 1.2; 95% CI, 0.12-11.87; *P* = .88), showed no significant difference in incomplete study rates. All dogs swallowed the device without difficulty, and signal transmission remained unaffected throughout the entire procedure. No dogs experienced adverse effects related to the administration of simethicone and endoscopic capsules after the procedure.

**Table 4:** Univariable logistic regression analysis of risk factors for incomplete study rates in 15 client-owned dogs undergoing 2 VCE procedures with different protocols.

	***n*, study**	**Incomplete study, *n* (%)**	**OR**	**95% CI**	***P*-value**
**Protocol**	Total 30	5 (17%)			
**Control (NS)**	15	3 (20%)	0.62	0.09–4.34	.63
**Simethicone**	15	2 (13%)			
	***n*, dog**	**1 ≤ incomplete (of 2), *n* (%)**	**OR**	**95% CI**	***P*-value**
**Sex**	Total 15	4 (27%)			
**Neutered male**	12	2 (17%)	10	0.58–171.20	.11
**Spayed female**	3	2 (67%)			
**Age (y)**	Total 15	4(27%)			
**≤7.67**	8	1 (13%)	5.26	0.40-68.95	.21
**7.67<**	7	3 (43%)			
**Weight (kg)**	Total 15	4			
**≤7**	8	2 (25%)	1.2	0.12–11.87	.88
**7<**	7	2 (29%)			

Abbreviations: CI = confidence interval; NS = normal saline; OR = odds ratio

## Discussion

Simethicone had a region-specific effect on VQ in the VCE. Simethicone administration significantly improved VQ in the small intestine, especially in the proximal segment, with all variables showing significant between-group differences. Simethicone administration did not have significant effects in the distal small bowel. These findings indicate that the benefit of simethicone during VCE in dogs is not uniform across GI regions, and emphasize the importance of segment-specific evaluation when assessing bowel preparation strategies.

Simethicone acts by decreasing the surface tension of intraluminal gas bubbles, allowing small bubbles to coalesce into larger ones that are more readily cleared from the GI tract.^14^^,^^16^^,^^20^ In human medicine, the use of anti-foaming agents such as simethicone during VCE is recommended to enhance VQ.^14^^,^^16–20^ However, there have been no veterinary studies evaluating the effects of simethicone on VQ during VCE. Moreover, most studies in humans reported the combination of simethicone with other purgatives, with only 1 study assessing its efficacy as a single agent.^16^ In that study,^16^ simethicone alone improved the VQ in the proximal small bowel, but not in the distal small bowel, which is consistent with our results. The authors suggested that this result may be because of a smaller amount of simethicone reaching the distal small intestine.^16^ Our findings may be explained by that same reasoning, yielding a more pronounced effect in the proximal segment.

The 2 individual variables (variables 1 and 2) were included to enhance the reliability of image assessment by incorporating complementary evaluation criteria that capture different aspects of VQ. Variable 1 reflects the proportion of mucosa visualized, whereas variable 2 reflects the degree of obscuration by bubbles, debris, blood, or bile, and therefore these variables are based on different criteria and are not identical measures. In our study, both variables demonstrated significant improvement in the entire and proximal small bowel, but variable 2 generally yielded lower scores than variable 1, likely because of its stricter evaluation criteria. Although both variables 1 and 2 demonstrated excellent intra-observer and inter-observer agreement, supporting the reliability of both scoring approaches, variable 2 may be more suitable for studies designed to evaluate the effects of anti-foaming agents, because it more directly assesses improvements in obscuration, including bubble-related obscuration.

Although the difference was not significant, VQ in the stomach tended to be lower with simethicone administration, which may be attributed to several anatomical and physiological features of the stomach. Unlike the small intestine, the stomach has a wider lumen, allowing for greater accumulation of intraluminal contents, including mucus, food debris, bile, and bubbles, which can obscure mucosal surfaces. Simethicone improves image clarity by decreasing the number of bubbles. However, we did not separately analyze the degree of obscuration caused by bubble formation and other interfering materials, which impedes determination of the specific contribution of simethicone to bubble clearance. Therefore, VQ in the stomach may not fully reflect the anti-foaming effect of simethicone. Moreover, the interaction of simethicone with other luminal contents may have contributed to increased turbidity or obscured mucosal surfaces. However, recent studies in humans have shown that administering simethicone before conventional endoscopy improves gastric visualization.^23^ Notably, conventional endoscopy involves active insufflation of the gastric lumen, offering a wide, controllable, real-time field of view. In contrast, capsule endoscopy captures images passively within a limited trajectory. These between-modality differences may account for these discrepancies regarding gastric visualization. Given the small sample size, wide CIs, and high inter-individual variability in gastric variables, whether this numerical trend reflects a true physiological effect or merely random variation remains unclear.

Although improved VQ is presumed to enhance diagnostic performance, evidence linking simethicone administration to increased diagnostic yield remains limited.^14^^,^^16–20^ One study in humans reported improved diagnostic yield in the proximal small bowel when simethicone was combined with mannitol alone.^17^ Similarly, there have been no veterinary studies on the effect of simethicone on diagnostic yield, emphasizing the need for further research to clarify its clinical utility in this field.

In our study, a fixed number of still images was extracted from each GI region to standardize VQ evaluation across all dogs. Initially, time-based extraction at fixed intervals (eg, 5-min interval) was considered.^15^ However, given the wide variability in GI transit times among individual dogs, this approach yields a highly inconsistent number of frames. Some cases had an insufficient number of available images for meaningful assessment, especially in regions with rapid transit. To address this limitation, we divided the total transit time by the required number of images for each region, allowing for the calculation of a tailored capture interval for each dog. Doing so ensured that all dogs contributed an equal number of images per GI region, which were evenly distributed throughout the transit period. This approach enhanced the consistency and reliability of the visualization scoring, regardless of individual differences in transit time. To validate the consistency of this approach, intra-patient agreement was assessed by repeatedly applying the same extraction method within individuals. We observed excellent intra-patient agreement, which supports the reproducibility and reliability of this standardized sampling approach. This finding indicates that our approach can be consistently applied across cases, regardless of individual differences in the GI transit time. However, whether this approach can fully represent the overall cleansing level of each GI segment in VCE in dogs requires further investigation.

Incomplete studies are a major limitation, because they restrict the comprehensive evaluation of the GI tract, and a method to improve completion rates exists. In human medicine, the overall rate of incomplete studies is 20%-30%. Prokinetic agents such as metoclopramide and erythromycin have been used to decrease CTT and improve completion rates, but their effects have been inconsistent.^14^^,^^24–29^

Previous veterinary studies have reported variable incomplete study rates ranging from 0% to 62%.^1^^,^^2^^,^^4^^,^^6^^,^^8–10^^,^^30^ In a recent study, authors compared macroscopic GI morphology between dogs with chronic enteropathy and healthy controls; all dogs successfully underwent VCE with no reported complications.^4^ All dogs in this study weighed >10 kg (median body weight, 25.9 kg; IQR, 20.6-30.9). A previous veterinary study reported an overall incompletion rate of 39% in dogs with questionable or overt GI bleeding.^10^ Another study reporting the feasibility and complications of VCE in dogs weighing ≤7 kg had a markedly high incomplete study rate (62%).^6^ In our study, the overall incomplete study rate was 17%, which is relatively low despite the inclusion of only small dogs, in which higher incomplete rates have been reported in previous studies. This outcome may be attributed to the exclusive enrollment of clinically healthy individuals and the use of a smaller capsule device, which may have facilitated gastric passage. Body size may influence capsule transit dynamics, particularly given the relatively large capsule-to-lumen size ratio in small dogs. The smaller capsule used in our study (23 × 9.5 mm) compared with previously available systems (approximately 31 × 11 mm) was specifically designed to address this constraint and to improve feasibility in dogs <10 kg, a population for which earlier, larger capsules were poorly suited. No significant between-protocol differences in completion rate, GTT, or SBTT were identified, suggesting that simethicone administration did not influence capsule transit under the conditions of our study. These results are consistent with previous studies in humans reporting that simethicone administration does not affect completion rates.^16^^,^^18^^,^^19^ Collectively, these findings highlight the substantial variability in incomplete study rates across veterinary VCE studies and indicate that further research is needed to better define the factors associated with capsule completion.

Regarding pre-procedure fasting protocols, we employed a minimum 12-hour fast before capsule administration. However, veterinary VCE studies have reported variable fasting durations ranging from 12 to 48 hours,^1^^,^^4^^,^^5^^,^^7^^,^^11^^,^^31^^,^^32^ and no universally accepted guideline currently exists for dogs. The manufacturer of the VCE system used in our study (Intromedics) recommends a minimum 12-hour fasting period, and previous veterinary studies using similar durations have reported acceptable image quality.^1^^,^^5^^,^^7^^,^^31^ Notably, VCE differs from conventional upper GI endoscopy in that it does not require general anesthesia and relies entirely on physiologic GI motility for capsule progression. Although the relationship between fasting duration and GI motility in dogs undergoing VCE has not been systematically evaluated, excessively prolonged fasting theoretically could decrease luminal stimulation and delay gastric emptying, potentially increasing incomplete study rates, a concern particularly relevant in small dogs.^33^ In our study, the standardized 12-hour protocol was applied consistently across all dogs, allowing valid internal comparison between protocols while maintaining reasonable gastric clearance. Nevertheless, future studies specifically investigating the optimal balance between adequate gastric clearance and preservation of physiologic motility would be valuable for establishing evidence-based veterinary VCE preparation guidelines.

In veterinary medicine, limited information is available regarding the appropriate dose of simethicone for VCE. We referenced studies in humans as well as veterinary case reports describing VCE use in a dog with acute hemorrhagic vomiting and chronic enteropathy.^5^^,^^7^^,^^14^^,^^16–20^ In veterinary case reports,^5^^,^^7^ simethicone was administered at a dose of 20 mg per dog to improve the image in capsule endoscopy without any assessment of its efficacy in enhancing VQ. Previous studies in humans analyzing the effect of simethicone on VQ used doses of 100 mg or 300 mg per person,^14^^,^^16^^,^^18–20^ with only 1 report using 800 mg with mannitol.^17^ In our study, all dogs received a fixed dose of 20 mg of simethicone, which is comparable to the dosage used in adult humans. Nevertheless, because fixed dosing resulted in substantial inter-individual variation in weight-adjusted exposure, ranging from 2.0 mg/kg (for a 10 kg dog) to 5.21 mg/kg (for a 3.84 kg dog), dose variability remains an important limitation when interpreting the effects of simethicone in our study. To assess whether this variability influenced study outcomes, we performed correlation analysis between weight-adjusted dose (mg/kg) and VQ difference between protocols across all GI segments and scoring variables. No significant associations were identified, and correlation coefficients were weak and inconsistent in direction. These findings suggest that variability in weight-adjusted dosing was unlikely to have substantially influenced visualization outcomes. However, given the small sample size, a potential dose–response relationship cannot be definitively excluded. Given the preliminary nature of our study, future research is warranted to determine the optimal dosage of simethicone for veterinary VCE as well as to explore alternative administration protocols, including different pre-examination fasting periods and the use of additional bowel preparation agents.

The clinical implications of our findings merit consideration. Simethicone offers a simple, well-tolerated approach that may improve VCE VQ without altering transit times or increasing incomplete study rates. Given the small sample size and preliminary nature of our study, these findings suggest that simethicone potentially could be considered as a premedication option to enhance small intestinal visualization during VCE in dogs, particularly in scenarios where a low-burden preparation is desired, such as in outpatient settings, stress-sensitive patients, or small dogs. However, these results should be interpreted with caution given our study’s limitations. Additional large-scale, adequately powered studies are warranted to confirm these findings, to determine the clinical relevance of the observed improvements, and to explore optimal dosing strategies and administration protocols for veterinary VCE.

Our study had some limitations. First, a sample size calculation was not performed because no prior veterinary data were available to estimate the expected effect size. These results may nonetheless provide preliminary reference values for future studies. Consequently, the relatively small sample size may have limited statistical power to detect modest effects, particularly in the subgroup analyses. Although significant between-group differences in VQ were observed in certain regions, wide CIs and borderline *P*-values in others suggested underpowering of the statistical analyses to confirm smaller but clinically relevant differences. Second, although the pre-procedure fasting period was standardized to at least 12 hours, the diet before fasting was not standardized. Accordingly, variability in dietary composition and digestibility may have influenced the nature of the residual gastric contents, which in turn may have affected the interaction between simethicone and luminal materials, such as mucus or bile. Third, free access to water was allowed throughout the procedure in accordance with ethical standards. However, uncontrolled differences in water intake may influence GI motility or intraluminal clarity, particularly in the stomach. These factors highlight the need for further controlled studies to optimize VCE preparation protocols and better understand the effects of simethicone across GI regions.

Simethicone administration significantly improved VQ in the small intestine, especially in the proximal segment, indicating a region-specific effect. These findings suggest that simethicone may be a useful premedication for enhancing small intestinal visualization during VCE in dogs. No significant between-protocol differences were observed in the gastric or SBTTs, as well as in incomplete study rates. Additional large-scale studies with various administration protocols are warranted to determine the optimal dosage of simethicone for veterinary VCE.

## Supplementary Material

7_Supporting_information_(table)_aalag183

## Off-label antimicrobial declaration

Authors declare no off-label use of antimicrobials.

## Institutional animal care and use committee or other approval declaration

Approved by the Institutional Animal Care and Use Committee of the Seoul National University (SNU-240102-4-4), and all procedures were conducted in accordance with relevant institutional and national guidelines for the care and use of laboratory animals. All owners signed a consent form before their dogs were enrolled.

## Abbreviations

BCS: body condition score
CTT: capsule transit time
GI: gastrointestinal
GTT: gastric transit time
ICC: intraclass correlation coefficient
IACUC: Institutional Animal Care and Use Committee
NS: normal saline
OR: odds ratio
SBTT: small bowel transit time
VCE: video capsule endoscopy
VQ: visualization quality
