# Supplementary material for: Effect of simethicone on visualization quality and capsule transit time during video capsule endoscopy in healthy dogs
Source: J Vet Intern Med. 2026 Aug 26;40(4):aalag183. doi: 10.1093/jvimsj/aalag183 (PMC13513761; doi:10.1093/jvimsj/aalag183)
Supplement: Supplementary file 1 [file 7_supporting_information_(table)_aalag183.docx]

| Supporting information  Table 1. Correlation between weight-adjusted simethicone dose (mg/kg) and between-protocol differences in visualization quality across gastrointestinal segments and parameters | | | | |
| --- | --- | --- | --- | --- |
|  | parameter | n | r | P-value |
| Stomach | 1 | 12 | -0.166 | 0.607 |
|  | 2 | 12 | -0.080 | 0.806 |
|  | 3 | 12 | -0.130 | 0.686 |
| Proximal small bowel | 1 | 11 | -0.216 | 0.524 |
|  | 2 | 11 | -0.348 | 0.294 |
|  | 3 | 11 | -0.295 | 0.379 |
| Distal small bowel | 1 | 11 | 0.221 | 0.514 |
|  | 2 | 11 | 0.249 | 0.459 |
|  | 3 | 11 | 0.236 | 0.485 |
| Entire small bowel | 1 | 11 | -0.003 | 0.992 |
|  | 2 | 11 | -0.239 | 0.479 |
|  | 3 | 11 | -0.132 | 0.700 |
| Note: Between-protocol differences were calculated as the mean VQ score for the control protocol minus that for the simethicone protocol. Correlation coefficients (r) describe the association between weight-adjusted simethicone dose (mg/kg) and the between-protocol difference in visualization quality. Parameter 1 indicates the proportion of mucosa visualized; Parameter 2, the degree of obscuration by bubbles, debris, blood, or bile; and Parameter 3, the mean of Parameters 1 and 2. | | | | |
